# PET/CT-Based Characterization of 18F-FDG Uptake in Various Tissues Reveals Novel Potential Contributions to Coronary Artery Disease in Psoriatic Arthritis

**DOI:** 10.3389/fimmu.2022.909760

**Published:** 2022-06-02

**Authors:** Daniella M. Schwartz, Philip Parel, Haiou Li, Alexander V. Sorokin, Alexander R. Berg, Marcus Chen, Amit Dey, Christin G. Hong, Martin Playford, McKella Sylvester, Heather Teague, Evan Siegel, Nehal N. Mehta

**Affiliations:** ^1^ National Institute of Allergy and Infectious Diseases, National Institutes of Health, Bethesda, MD, United States; ^2^ Division of Rheumatology and Clinical Immunology, University of Pittsburgh School of Medicine, Pittsburgh, PA, United States; ^3^ National Heart Lung and Blood Institute, National Institutes of Health, Bethesda, MD, United States; ^4^ Department of Internal Medicine, Georgetown University Medical Center, Washington, DC, United States; ^5^ Arthritis and Rheumatism Associates, Wheaton, MD, United States

**Keywords:** psoriasis, psoriatic arthritis, cardiovascular disease, atherogenesis, PSA, imaging

## Abstract

**Background and Objectives:**

Psoriasis is a heterogeneous inflammatory disease that involves the skin, joints, liver, heart, and other organs. Psoriatic arthritis (PsA) is associated with cardiovascular disease (CVD), but the relative contributions of inflammatory and metabolic dysregulation to CVD are incompletely understood. We set out to discover novel potential contributors to CVD in PsA patients by comprehensively phenotyping a cohort of PsA patients using these advanced technologies.

**Methods:**

In this cross-sectional analysis of a cohort study, we investigated associations of systemic inflammation and metabolic dysregulation with Coronary CT angiography (CCTA)-proven coronary artery disease (CAD) in 39 subjects with PsA. We measured traditional CVD risk factors [blood pressure, Body Mass Index (BMI), diabetes, age, sex, smoking], serum markers of systemic inflammation (hsCRP, GlycA) and metabolic dysfunction (cholesterol efflux capacity), and inflammatory cytokines (IL-1β, IL-6, IL-12/IL-23, IL-17A, TNF-α, IFN-γ). We also incorporated radiographic measures of metabolic dysfunction (visceral and subcutaneous adipose volume) and tissue-specific inflammation (positron emission tomography-computed tomography, PET-CT). To quantify relative contributions of FDG (fluorodeoxyglucose) uptake and adiposity to coronary plaque, we performed multiple linear regression, controlling for Framingham risk score (FRS) and FRS + visceral adiposity.

**Results:**

Compared with non-psoriatic volunteers, subjects with PsA had elevated markers of metabolic and inflammatory disease, which was more pronounced in subjects with moderate-to-severe skin disease. This included visceral (p = 0.005) and subcutaneous (p = 0.004) adiposity, BMI (p = 0.001), hemoglobin A1C (p = 0.037), high sensitivity C-reactive protein (p = 0.005), IL-6 (p = 0.003), IFN-γ (p = 0.006), and liver FDG uptake (p = 0.03). In subjects with PsA, visceral adiposity correlated significantly with subclinical CAD (standardized β = 0.681, p = 0.002), as did FDG uptake in bone marrow (standardized β = 0.488, p = 0.008), liver (standardized β = 0.619, p < 0.001), spleen (standardized β = 0.523, p = 0.004), and subcutaneous adipose (standardized β = 0.524, p = 0.003).

**Interpretation:**

Together, these findings reveal inflammatory and metabolic potential contributors to subclinical CAD in PsA, including adipose inflammation, and suggesting novel targets for CVD prevention and treatment in PsA.

## Introduction

Psoriasis is a complex disease with substantial clinical heterogeneity. In addition to skin inflammation, psoriasis can manifest with axial or peripheral arthritis (PsA), liver disease, lung disease, gastrointestinal inflammation, aortic root involvement, or nail disease (1). Cardiovascular disease (CVD) is the leading cause of death in patients with psoriasis and PsA and is often regarded as a component of the clinical spectrum encompassed by psoriatic disease (1, 2). However, the factors driving psoriatic atherogenesis are incompletely understood. Psoriatic diseases are associated with high rates of traditional CV risk factors (2). Systemic inflammation, which is often more severe in PsA, also promotes atherogenesis (2, 3). Additionally, psoriatic diseases –particularly PsA – are associated with metabolic dysregulation including visceral adipose tissue expansion and HDL dysfunction, leading to impaired cholesterol metabolism (4, 5). Metabolic dysregulation interacts with inflammation to promote atherosclerosis through as-yet unknown mechanisms (5, 6). Defining these mechanisms and interactions can address a major unmet need by identifying factors that can be targeted to prevent the high rates of CVD in patients with psoriatic diseases.

Traditionally, systemic inflammation is evaluated by clinical laboratory measurement of acute phase reactants, mainly high sensitivity C-reactive protein (hs-CRP) (7). More recently, 18-fluorodeoxyglucose positron emission tomography computed tomography (FDG-PET-CT) has been used to sensitively and noninvasively quantify systemic and tissue inflammation (3). Together with serum cytokine and glycoprotein acetylation (GlycA) levels, FDG-PET-CT can provide a more complete picture of inflammatory disease pathogenesis than hs-CRP alone (3, 6). Similarly, metabolic dysregulation is usually estimated using traditional CV risk factors but can be more sensitively quantified by incorporating radiographic measures of adiposity and advanced lipid phenotyping assays like cholesterol efflux capacity (8). Combined with coronary CT angiography (CCTA), a noninvasive angiographic technique that provides a quantifiable measure of coronary artery disease (CAD), these techniques allow for a multimodal real-time evaluation of potential contributors to psoriatic atherogenesis (9).

We hypothesized that combining CCTA with other advanced radiographic and serum-based assays might identify novel contributions to subclinical coronary disease in PsA (2). We performed a cross-sectional analysis of a prospective cohort study, utilizing data from a one-time initial visit. We measured metabolic dysregulation, inflammatory burden, and subclinical CAD in subjects with PsA and age-sex matched non-psoriatic volunteers, utilizing traditional clinical disease measures, serum-based assays, CCTA, and FDG-PET-CT.

## Methods

### Study Design and Population

**Figure 1:**
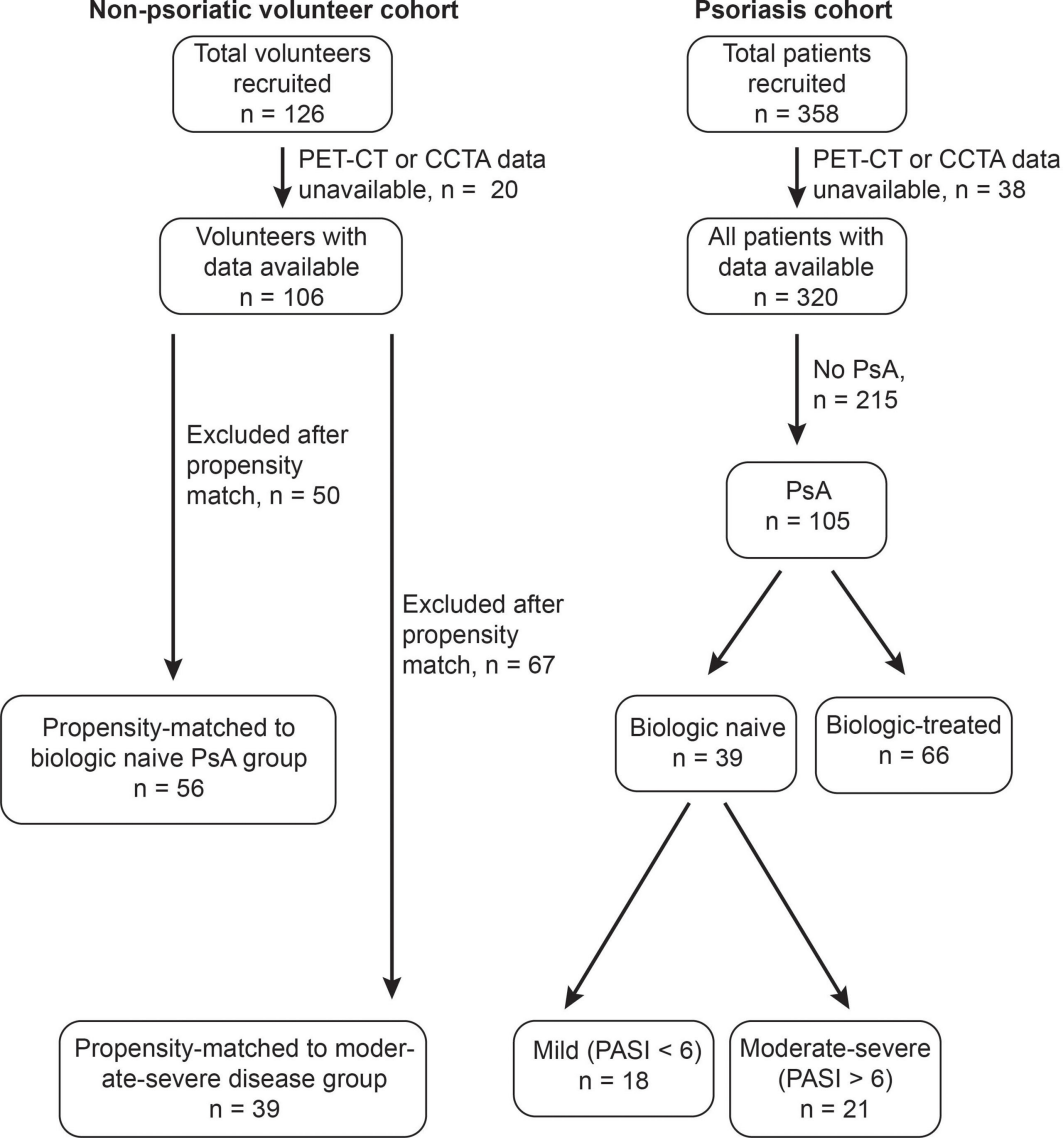
Identification of subjects with treatment-naive psoriatic arthritis (PsA) within a cohort of patients with psoriatic diseases, and of age-sex-matched non-psoriatic volunteers. 358 total patients with psoriatic diseases were recruited, of whom 320 were confirmed to have psoriatic disease and had PET-CT and CCTA data available. Of these 105 had psoriatic arthritis, 39 of whom were biologic-naive. 21 biologic naive patients with PsA had moderate-severe skin disease. 126 total non-psoriatic volunteers were recruited, of whom 106 had PET-CT and CCTA data available. 56 volunteers were age-sex-matched to the full treatment-naïve PsA cohort, whereas 39 volunteers were age-sex-matched to the 21 subjects with moderate-severe skin disease.

358 subjects with psoriasis were recruited between Jan 2013-May 2021 as part of an IRB-approved prospective cohort study at the NIH Clinical Center (**Figure 1**, 13H-0065). Full inclusion and exclusion criteria were previously reported (10). 204 subjects who had received biologic disease modifying antirheumatic drugs (bDMARDs) were excluded. Age and sex matched volunteers were recruited through a separate IRB approved protocol at the NIH Clinical Center (NCT01934660). All subjects provided written informed consent.

### Clinical Assessment

Full details of clinical assessment were previously described (10). Briefly, patients underwent evaluation by trained providers at the NIH Clinical Center to verify psoriasis onset, duration, and severity (Psoriasis Area and Severity Score, PASI). Psoriatic arthritis (PsA) was diagnosed by a rheumatologist based on diagnosis of psoriasis and seronegative inflammatory arthritis; all patients satisfied CASPAR criteria. Joint disease activity was assessed systematically by a rheumatologist in a subset of patients (n = 16). Clinical assessments included full medical histories, physical examinations, PASI quantification, medication evaluations, anthropometric measurements, and clinical laboratory assays.

### Imaging Assessment

All patients underwent CCTA using a 320-detector row Aquilion ONE ViSION (Toshiba); acquisition details were previously described (11). All scans were analyzed in a blinded fashion using QAngio CT (Medis). FDG-PET-CT imaging was performed using a Biograph mCT PET-CT 64-slice scanner (Siemens Medical Solutions). After an overnight fast (≥8 hours), images were acquired approximately 60 minutes (62 ± 1 minutes) after administration of FDG (10 mCi). All patients underwent identical PET-CT protocols with the same team of technologists. Standard bed (3 min each, scanning cranially to caudally) were obtained for each patient using 1.5-mm axial slices. Patients with fasting glucose >200 mg/dl were excluded. Analysis was done in a blinded fashion using dedicated PET-CT image analysis software (OsirixTM, Pixmeo SARL) as previously described (11). Visceral and subcutaneous adipose tissue volumes were analyzed using low-dose CT: 100 transverse slices from the caudal sternum to cranial pubic symphysis were interpreted using automated software with contour model algorithm. Visceral and subcutaneous adipose tissue volumes were demarcated as previously described (11).

### Serum and Blood Laboratory Assessment

Clinical laboratory assays including GlycA and serum lipid levels were measured at the NIH Clinical Center. Serum cytokines were measured using multiplex ELISA (Mesoscale). Cholesterol efflux capacity was measured using a cell-based assay in J774 macrophages as previously described (9).

### Statistical Analysis

PsA and non-psoriatic volunteers (controls) were age- and sex-matched as follows: non-psoriatic volunteers were matched in a 1:2 ratio according to propensity scores (age, sex) with a tolerance level of 0.2 to maximize sample size. Because the volunteer cohort is younger and contains more female subjects than the PsA cohort, we used a logistic regression model to perform matching. For matched data (**Table 1** and **Supplementary Table 1**), baseline characteristics are presented mean ± SD for parametric variables, median (interquartile range [IQR]) for nonparametric variables, and percentages (%) for categorical variables. Data were assessed for normality *via* Shapiro-Wilks test. Statistical significance was assessed by Student t-test for parametric variables, Wilcoxon rank-sum test for nonparametric variables, and Pearson’s χ2 test or Fisher-exact test for categorical variables. The moderate-severe psoriasis subgroup analysis was performed by identifying patients with PASI score >6, which was the median value for this cohort (4), and repeating propensity score matching. To quantify relative contributions of FDG uptake and adiposity to coronary plaque, we performed multivariate linear regression for each PET-CT parameter, with Framingham risk score (**Table 2**) or Framingham risk score and visceral adiposity (**Supplementary Table 2**) as predictors and non-calcified coronary artery burden (NCB) as the outcome. Standardized ß coefficients, coefficient of partial determination (partial R^2^), and P-values were compared. All statistical analyses were performed using Stata 17 (StataCorp) and R 4.0.5. A two-tailed P-value ≤ 0.05 was considered significant.

**Table 1:** Clinical, laboratory, immunological and imaging characteristics of biologic naïve subjects with PsA and moderate to severe skin disease (PASI > 6) and non-psoriatic volunteers.

Parameter	PsA (n = 21)	NPV (n = 39)	P-value
**Clinical Characteristics**			
Age (years)	54 (50 – 60)	53 (47 - 58)	0.852
Sex (male)	13 (62%)	27 (69%)	0.774
Framingham 10-Year Risk Score	3.2 (1.5 – 6.8)	2.9 (1.0 - 7.4)	0.570
Type 2 Diabetes Mellitus	6 (29%)	3 (11%)	0.146
Hyperlipidemia	6 (29%)	14 (50%)	0.224
Current smoker	5 (24%)	1 (4%)	0.072
Hypertension	4 (19%)	10 (36%)	0.338
Statin Use	5 (24%)	8 (29%)	0.963
DMARD Use	5 (24%)	**-**	**-**
NSAID Use	5 (24%)	–	–
**BMI (kg/m^2^)**	**33.3 (29.2 – 36.7)**	**27.2 (24.5 - 30.2)**	**0.001**
Waist-to-hip Ratio	0.97 (0.93 – 1.02)	0.96 (0.92 - 0.99)	0.284
Systolic blood pressure (mm Hg)	121.67 ± 13.64	117.56 ± 14.02	0.277
Diastolic blood pressure (mm Hg)	71.95 ± 10.34	71.51 ± 11.30	0.88
PASI score	8.6 (6.6 – 9.0)	**-**	**-**
DAPSA score (n = 8)	12.8 (7.8 – 41.1)	**-**	**-**
Psoriasis Disease Duration (years)	30 (19 – 35)	–	–
Total Body Surface Area Index	10 (5.8 – 13.7)	**-**	**-**
**Clinical Laboratory values**			
Total cholesterol (mg/dL)	184.81 ± 30.13	184.47 ± 49.88	0.975
HDL cholesterol (mg/dL)	50 (44 – 57)	49 (42 - 68)	0.816
LDL cholesterol (mg/dL)	107 (93 – 118)	111 (64 - 131)	0.624
Triglycerides (mg/dL)	114 (94 – 180)	101 (69 - 152)	0.23
**hs-CRP** (mg/L)	**3.8 (1.6 - 7.1)**	**1.3 (0.9 - 1.9)**	**0.005**
Cholesterol efflux capacity	0.95 (0.84 - 1.05)	1.01 (0.90 - 1.16)	0.131
**GlycA** (µmol/L)	**420 (381 - 464)**	**333 (314 - 360)**	**<0.001**
**Hemoglobin A1C**	**5.7 (5.5 - 5.9)**	**5.4 (5.1 - 5.7)**	**0.037**
**Serum cytokines (pg/mL)**			
IL-1β (N = 17, 24)	1.87 (0.62 – 2.60)	–	–
**IL-6 (N = 17, 27)**	**1.51 (0.84 – 3.57)**	**0.83 (0.51 - 1.22)**	**0.003**
IL-12/IL-23 (N = 5, 9)	77.88 (51.36 - 91.39)	69.82 (38.94 - 101.46)	0.679
IL-17A (n = 17, 23)	1.31 (0.42 - 3.37)	0.79 (0.53 - 1.64)	0.376
TNF-α (n = 17, 27)	1.45 (0.67 - 2.85)	1.26 (0.49 - 1.54)	0.921
**IFN-γ (n = 17, 27)**	**8.00 (6.39 - 21.60)**	**3.72 (2.47 - 7.09)**	**0.006**
**CT**			
**Visceral Adipose Tissue (cc)**	**22743 (17206 – 25981)**	**12044(7410 - 21446)**	**0.005**
**Subcutaneous Adipose Tissue (cc)**	**22523 (16883 - 29569)**	**14188 (10503 - 18647)**	**0.004**
**PET-CT**			
Aortic vascular (TBR)	1.81 (1.71 - 1.90)	1.75 (1.70 - 1.86)	0.498
Bone Marrow (SUV_max_)	4.38 (3.34 - 6.09)	3.57 (3.10 - 4.02)	0.074
**Liver (SUV_max_)**	**5.44 (5.02 - 7.25)**	**4.55 (4.12 - 5.16)**	**0.03**
Spleen (SUV_max_)	4.24 (3.26 - 5.95)	3.55 (3.22 - 3.73)	0.261
Subcutaneous Adipose Tissue (SUV_max_)	0.61 (0.47 - 0.74)	0.57 (0.43 - 0.63)	0.282
**CCTA (per artery)**	**PsA** **(n = 63 arteries)**	**NPV** **(n = 117 arteries)**	
**TB (x100)**, mm^2^	**1.47 ± 0.66**	**1.11 ± 0.39**	**0.001**
**NCB (x100)**, mm^2^	**1.42 ± 0.62**	**1.07 ± 0.40**	**<0.001**

PsA, psoriatic arthritis; NPV, non-psoriatic volunteers; cDMARD, conventional disease modifying antirheumatic drugs; NSAIDs, nonsteroidal anti-inflammatory drugs; BMI, body mass index; PASI, psoriasis area severity index; BSA, body surface area; HDL, high density lipoprotein; LDL, low density lipoprotein; CRP, C-reactive protein; GlycA, glycoprotein acetylation; IL-, interleukin-; IFN, interferon; TBR, target-to-background ratio; CT, computed tomography; PET-CT, positron emission tomography; SUV_max_, maximal standardized uptake value; CCTA, coronary artery CT angiography; TB, total coronary artery burden; NCB, non-calcified coronary artery burden.

Bold, statistically significant.

**Table 2:** Regression analysis of systemic inflammation and fat variables with NCB in biologic-naïve subjects with PsA (n = 34), adjusted for Framingham Risk Score.

Variable of interest	*β* estimate	Standardized *β* estimate	Partial *R* ^2^	P-value
**Bone marrow SUV**	**0.157**	**0.488**	**23.0**	**0.008**
**Liver SUV**	**0.176**	**0.619**	**39.7**	**<0.001**
**Spleen SUV**	**0.217**	**0.523**	**27.3**	**0.004**
**Subcutaneous Fat SUV**	**0.907**	**0.524**	**28.0**	**0.003**
Aortic vascular TBR	0.651	0.275	3.6	0.249
**Visceral Adipose Tissue (cc)**	**<0.001**	**0.681**	**28.8**	**0.002**
Subcutaneous Adipose Tissue (cc)	<0.001	0.301	6.4	0.107

NCB, non-calcified coronary artery burden; PsA, psoriatic arthritis; SUV, standardized uptake value; TBR, target-to-background ratio. Bone marrow (partial R^2^ = 23, p = 0.008), spleen (partial R^2^ = 39.7, p < 0.001), and liver (partial R^2^ = 27.3, p = 0.004) SUV correlated significantly with NCB. FDG uptake in all three tissues is associated with systemic inflammation; hence, this finding suggests that systemic inflammation contributes to CAD in PsA. Findings were similar for visceral adipose tissue volume (partial R^2^ = 28.8, p = 0.002), likely reflecting the role of metabolic dysregulation in psoriatic CAD. Unexpectedly, aortic vascular target-to-background ratio (TBR) had no significant correlation with NCB (partial R^2^ = 3.6, p = 0.249), despite previous reports that joint involvement correlates with vascular inflammation in psoriasis (3). Conversely, subcutaneous adipose SUV was significantly associated with NCB (partial R^2^ = 28, p = 0.003). Visceral adiposity is itself a cause of systemic and adipose inflammation; we therefore adjusted for this parameter and reanalyzed associations with FDG uptake. Liver (partial R^2^ = 21.4, p = 0.013), spleen (partial R^2^ = 13.4, p = 0.043), and subcutaneous fat (partial R^2^ = 16.6, p = 0.027) FDG uptake were still significantly associated with NCB after adjusting for visceral adiposity (**Supplementary Table 2**).

bold, statistically significant.

## Results

### Demographics and Disease Assessments of the PsA and Volunteer Cohorts

**Figure 2:**
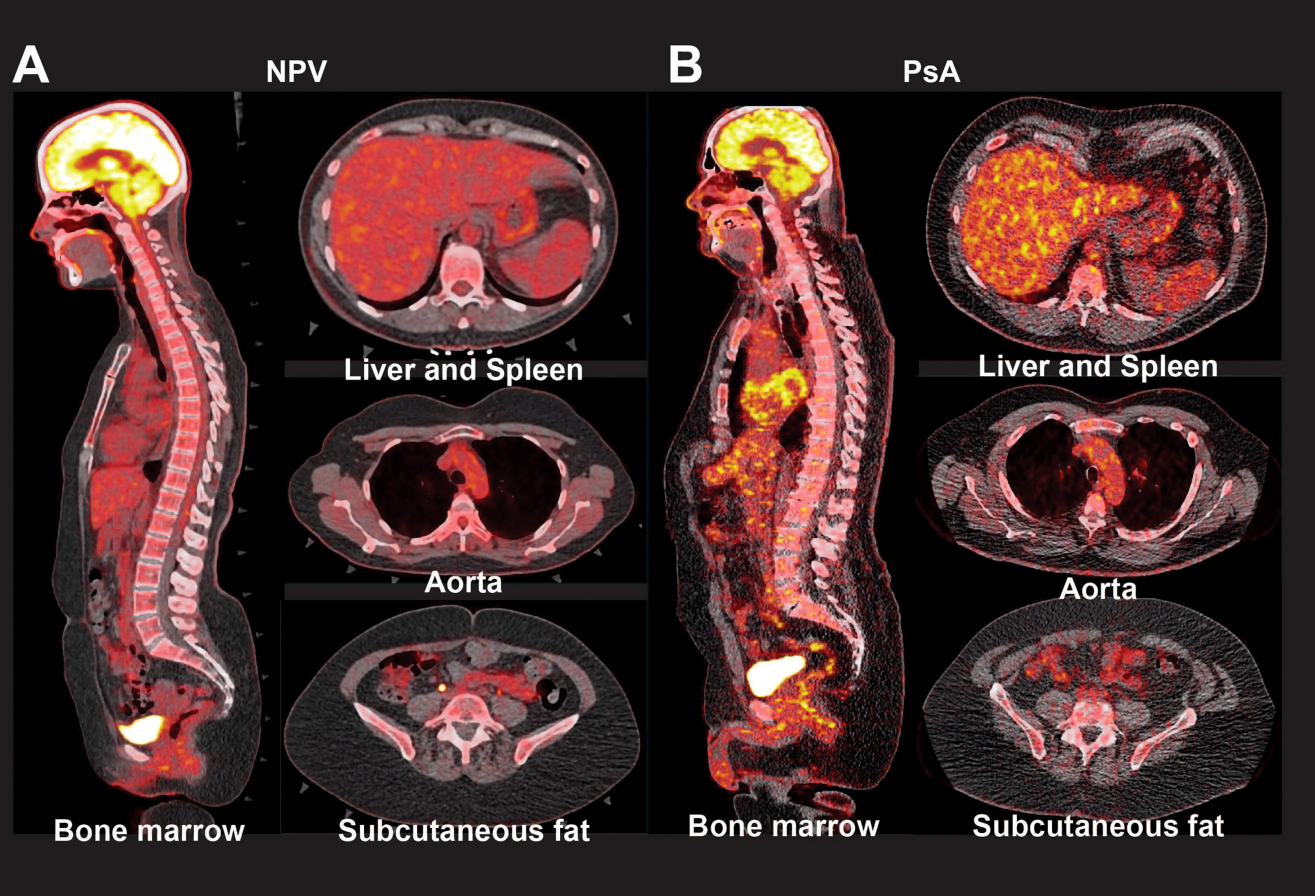
Radiographic markers of inflammation in patients with psoriatic arthritis. Representative PET-CT images show FDG uptake in the bone marrow, liver, spleen, aorta, and subcutaneous fat of non-psoriatic volunteers (NPV, **A**), and patients with psoriatic arthritis (PsA, **B**).

39 subjects with biologic-naïve PsA were compared with 56 age-sex-matched non-psoriatic volunteers (**Figure 1**). Because patients with severe psoriasis are at an increased risk of CVD, we separately analyzed clinical, laboratory and radiographic characteristics of this subgroup (1). Clinical, immunological, and demographic characteristics of subjects with moderate-severe skin disease (PASI > 6, the median value for this cohort that has been previously used as a cutoff for moderate-severe disease (4)) are shown in **Table 1**, while characteristics of the full treatment-naïve PsA cohort are shown in **Supplementary Table 1**. Representative CCTA and FDG-PET-CT images are shown in **Figures 2** **,** **3**.

**Figure 3:**
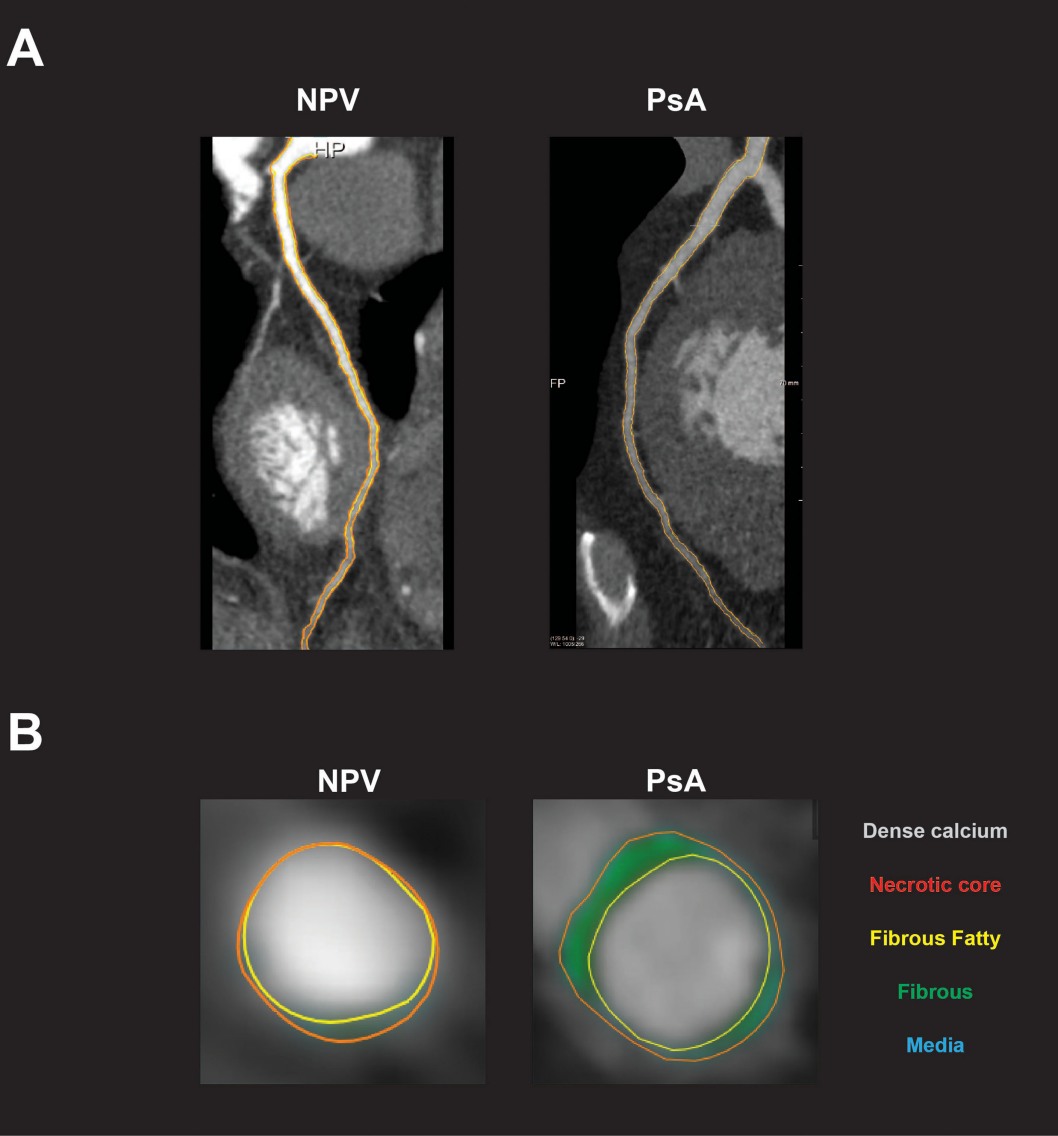
Subclinical coronary artery disease in patients with psoriatic arthritis. **(A)** Representative coronary artery CT angiogram (CCTA) images show left anterior descending (LAD) artery in non-psoriatic volunteers (NPV) and patients with psoriatic arthritis (PsA). **(B)** Representative CCTA images show coronary artery plaque in NPV and PsA. Total burden is determined by measuring the total plaque area (orange) around the lumen (yellow). Non-calcified coronary artery burden (NCB) is determined by calculating the difference between total and dense calcified burden. For these subjects, NCB x 100 = 0.602 (NPV) and 2.108 (PsA).

### Clinical, Laboratory and Radiographic Characteristics of the Full Treatment-Naïve PsA Cohort

Disease-modifying antirheumatic drug (DMARD) and non-steroidal anti-inflammatory drug (NSAID) were both used in PsA, consistent with the use of these agents for joint pain and inflammation (12). PASI scores and total BSA indices suggested a moderate degree of skin disease, and DAPSA scores suggested a mild-to-moderate amount of joint disease. Both IL-6 and hs-CRP – an IL-6-dependent acute phase reactant – were elevated in PsA (7). IFN-γ levels were also elevated in PsA, as was GlycA, a more comprehensive inflammatory marker. PET maximal standardized uptake values (PET-SUV_max_) quantify FDG uptake, which provides a radiographic measure of inflammation within tissues (3, 11). Bone marrow, liver, and spleen FDG update are associated with systemic inflammation; bone marrow and liver PET-SUV_max_ were significantly elevated in PsA (3, 11).

Because metabolic dysregulation promotes atherogenesis, we also compared clinical and measures of metabolic disease in PsA *vs*. in volunteers (13). Subjects with PsA had elevated systolic blood pressures (SBP) and a trend towards higher body mass index (BMI) relative to non-psoriatic volunteers. Because overall and visceral adiposity are associated with metabolic syndrome, we measured these parameters radiographically (14). Subjects with PsA had significantly elevated subcutaneous adiposity compared to volunteers, and a trend towards increased body mass index (BMI) and visceral adiposity.

### Clinical, Laboratory and Radiographic Characteristics of PsA Patients With Moderate-Severe Skin Disease

As in the general biologic-naïve cohort, subjects with PsA and moderate-severe skin disease had elevated GlycA, IFN-γ, hs-CRP and IL-6. PsA was more strongly associated with metabolic dysregulation in this subgroup than in the general cohort. BMI, visceral adiposity, and subcutaneous adiposity were significantly higher in PsA than in non-psoriatic volunteers. Hemoglobin A1c was also increased in PsA relative to volunteers, and there was a trend towards increased prevalence of type 2 diabetes mellitus.

CCTA reliably and noninvasively quantifies CAD, and psoriasis increases both total (TB) and non-calcified (NCB) coronary artery burden (9). In our cohort, TB and NCB were numerically increased in subjects with PsA relative to non-psoriatic volunteers, although differences were not statistically significant. In patients with moderate-severe skin disease, TB and NCB were significantly increased in PsA relative to volunteers.

### Associations of FDG Update With Non-Calcified Coronary Artery Burden in PsA

Having characterized the radiographic measures of inflammatory and metabolic dysregulation in PsA, we next investigated associations of these factors with CAD in patients with PsA. One way to determine the contribution of a factor to CAD is to calculate the partial correlation (*R* ^2^) and standardized β estimates of that variable with NCB (3, 4, 13, 15–17). We therefore measured the partial *R* ^2^ and standardized β of tissue-specific FDG-PET SUV with NCB. Values are shown in **Table 2**.

## Discussion

PsA is a major CVD risk factor, but the exact pathophysiologic mechanisms are poorly understood, presenting a challenge to prevention and treatment (1, 3, 9, 13). Here, we used conventional and novel techniques to measure subclinical CAD, metabolic dysregulation, systemic inflammation, and tissue-specific FDG uptake in patients with PsA. PsA was characterized by metabolic dysregulation, systemic inflammation, and subclinical CAD compared to age-sex-matched volunteers. All three were worse in subjects with moderate-severe skin disease, suggesting that the combination of severe skin inflammation and joint disease may be especially atherogenic. Factor analysis revealed multiple potential contributors to CAD in PsA including visceral adiposity and FDG uptake in the liver, spleen, bone marrow, and fat, but not in the aorta.

A central finding of this study is that PsA is an atherogenic state. The association of psoriasis with CVD is well-described, but studies investigating the exact risks of and contributions to CVD in PsA have had disparate results (1, 2, 8). This may be because previous studies have used different outcome measures including traditional CV risk factors, major cardiovascular events (MACE), and carotid intimal medial thickness (cIMT). We employed CCTA, a direct noninvasive angiographic measurement of CAD that allowed us to quantify early disease more accurately, sensitively, and directly than a late binary outcome like MACE or an indirect measurement like cIMT (18–20). Another potential reason for conflicting results of prior studies is that PsA often treated with biologics (2). Because treatment with biologics can prevent psoriatic CVD, this could have affected results of previous studies (12). We avoided this potential confounder by investigating only biologic-naïve subjects.

Atherogenesis is a complex process with multiple drivers including metabolic dysregulation and systemic inflammation (10, 14). Psoriasis is an inflammatory disease that classically causes skin pathology but is clinically heterogeneous and can include systemic inflammation, as evidenced by elevated serum GlycA, hs-CRP, and cytokine levels. In our cohort, measures of IL-6-associated systemic inflammation were elevated in PsA. The IL-6 inhibitor clazakizumab has been shown to modestly improve musculoskeletal manifestations of PsA, but to have no effect on skin disease, so that its development was not pursued for PsA (21). Together with our data, this reported efficacy of clazakizumab for musculoskeletal psoriatic disease suggests that IL-6 may be a PsA-specific inflammatory mediator that is not as important for cutaneous inflammation. PsA is also associated with increased adiposity in population studies, including in this cohort, and adiposity can itself cause of elevated IL-6 and CRP (22). Because an extensive body of literature links IL-6-driven inflammation to CVD, targeting IL-6 may be a potential strategy to prevent PsA-associated CVD (7). Future studies will be needed to explore the underlying etiology of IL-6-driven inflammation and clinical implications in subjects with PsA.

Psoriasis also has strong associations with metabolic syndrome, although the reasons for this are poorly understood. In our cohort, PsA was significantly associated with visceral adiposity, subcutaneous adiposity, and glucose intolerance – particularly in subjects with moderate-severe skin disease. Increased adiposity may result from expansion of cutaneous inflammation to other compartments including the joints, intestine, fat, and liver. Future studies will be needed to determine whether visceral or subcutaneous adiposity promote extracutaneous psoriatic disease, including PsA. Notably, adipose tissue may itself serve as an inflammatory nidus in CVD pathogenesis. Previous studies have reported that psoriatic adipose tissue is infiltrated by immune cells, and that adipokine concentrations are elevated in PsA (5, 6). Accordingly, our regression analysis suggested that adipose inflammation likely contributed to CAD in PsA. We have previously reported that skin inflammation and sacroiliitis are both associated with aortic vascular inflammation, which is a known manifestation of psoriasis; however, our regression analysis suggests that aortic vascular inflammation was not associated with subclinical CAD in PsA (3). Future work will be needed to investigate the reasons for these associations, as well as the effects of bDMARD therapy on organ-specific inflammation. This will help determine whether targeting radiographic adipose inflammation might prevent atherogenesis in PsA.

One important limitation is the size of the treatment-naïve cohorts, which limited our power to detect differences between PsA and volunteers. However, the study was powered to detect significant differences in multiple parameters, particularly in the smaller group of subjects with moderate-severe skin disease. These variables included NCB, skin and systemic inflammation, visceral and subcutaneous adiposity, and other CVD risk factors.

Another limitation is that patients were not uniformly assessed for joint disease activity using clinical measures (i.e., DAPSA). We could not quantify arthritis disease activity radiographically because our PET/CT protocol uses planar imaging, which is not sensitive enough to reliably detect joint inflammation. We were therefore unable to measure the potential contribution of joint inflammation to subclinical CAD in our cohort using clinical or radiographic measures. Multiple studies suggest that joint and nail inflammation may contribute to CVD in patients with PsA (23–25). However, CVD is also linked to multiple other risk factors in patients with psoriatic disease (26, 27). One such risk factor is systemic inflammation: hsCRP and GlycA are elevated in many patients with psoriatic disease and correlate with subclinical CAD but do not usually correlate with joint disease activity (26, 27). Moreover, psoriatic inflammation can involve multiple organs including the aortic root, liver, eye, gastrointestinal tract, and adipose tissue (1). For this reason, it is important to also think about inflammation outside of the skin, joint, and nails when considering potential drivers of CVD in patients with PsA. Although we could not assess the potential contribution of joint inflammation to CVD, our approach did identify several other tissue-specific loci of inflammation that might promote subclinical CVD in PsA. Further studies will be needed to investigate the roles of these different inflammatory foci to CVD pathogenesis in patients with PsA relative to the contributions of skin, joints, and nail inflammation.

A final limitation is that DMARDs and NSAIDs were both used in PsA, which could have affected the risk of CAD PsA relative to non-psoriatic volunteers. However, clinical practice guidelines for PsA recommend both DMARD and NSAID therapy (12). Thus, these treatments reflect real-world PsA management that affects the relative risk of CAD. Important strengths include the specific focus on treatment-naïve subjects, high accuracy of clinical phenotyping, inclusion of mechanistic inflammatory and metabolic biomarkers, and use of cutting-edge imaging studies. Together, these allowed us to identify novel potential inflammatory and metabolic contributors to atherogenesis in PsA, representing an important advance in the field.

In conclusion, psoriasis is atherogenic, dysmetabolic, and inflammatory state, which is most pronounced in patients with moderate to severe skin disease. IL-6-driven systemic inflammation is increased in patients with PsA, providing a potential mechanistic link to atherogenesis in this population. Systemic inflammation is associated with CVD in PsA, suggesting that targeting IL-6 might be effective to prevent CVD in this population. Visceral adiposity is also associated with PsA-related CVD, as is adipose inflammation, suggesting a potential link between metabolic dysregulation and systemic inflammation. Future studies will be needed to follow up on the significance of this finding and to determine the effects of targeting these potential contributors to CVD in patients with PsA.

## Data Availability Statement

The raw data supporting the conclusions of this article will be made available by the authors, without undue reservation.

## Ethics Statement

This study was approved by the Institutional Review Board of the National Heart Lung and Blood Institute, NIH. The patients/participants provided their written informed consent to participate in this study.

## Author Contributions

Data collection: AB, AS, MC, CH, MP, and HT; Data analysis: DS, PP, and HL; Writing: DS, ES, MS, and NM. All authors contributed to the article and approved the submitted version.

## Funding

This work was funded by the intramural research programs of NIAID and NHLBI, and by the National Psoriasis Foundation.

## Conflict of Interest

NM has served as a consultant for Amgen, Eli Lilly, and Leo Pharma, receiving grants and other payments.

The remaining authors declare that the research was conducted in the absence of any commercial or financial relationships that could be construed as a potential conflict of interest.

## Publisher’s Note

All claims expressed in this article are solely those of the authors and do not necessarily represent those of their affiliated organizations, or those of the publisher, the editors and the reviewers. Any product that may be evaluated in this article, or claim that may be made by its manufacturer, is not guaranteed or endorsed by the publisher.
