# Supplementary material for: PET/CT-Based Characterization of 18F-FDG Uptake in Various Tissues Reveals Novel Potential Contributions to Coronary Artery Disease in Psoriatic Arthritis
Source: Front Immunol. 2022 Jun 2;13:909760. doi: 10.3389/fimmu.2022.909760 (PMC9201918; doi:10.3389/fimmu.2022.909760)
Supplement: Supplementary file 1 [file Table_1.docx]

**Table 1. Clinical, Laboratory, Immunological and Imaging characteristics of subjects with biologic-naïve PsA and non-psoriatic volunteers.**

| **Parameter** | **PsA**  **(n = 39)** | **Non-psoriasis volunteers**  **(n=56)** | **p-value**  **PsA vs. NPV** |
| --- | --- | --- | --- |
| **Clinical Characteristics** |  |  |  |
| Age (years) | 54 (49 – 59) | 53 (47- 58) | 0.617 |
| Sex (male) | 23 (59%) | 38 (68%) | 0.502 |
| Framingham 10-y Risk Score | 3.0 (1.3 – 6.8) | 2.4 (1.0 - 7.3) | 0.675 |
| Type 2 Diabetes Mellitus | 6 (15%) | 4 (10%) | 0.735 |
| Hyperlipidemia | 12 (31%) | 16 (41%) | 0.479 |
| Current smoker | 6 (15%) | 2 (5%) | 0.263 |
| Hypertension | 7 (18%) | 11 (28%) | 0.420 |
| Statin Use | 9 (23%) | 11 (28%) | 0.795 |
| cDMARD Use | 11 (28%) | - | **-** |
| NSAID Use | 12 (31%) | - | - |
| BMI (kg/m^2^) | 29.2 (26.1 – 34.5) | 27.4 (24.5 - 30.0) | 0.050 |
| Waist-to-hip Ratio | 0.95 (0.90 – 1.00) | 0.95 (0.90 - 0.99) | 0.562 |
| **Systolic blood pressure (mm Hg)** | **122.34 ± 12.30** | **115.29 ± 14.35** | **0.013** |
| Diastolic blood pressure (mm Hg) | 72.79 ± 9.95 | 70.66 ± 10.27 | 0.318 |
| PASI score | 6.0 (2.5 – 8.6) | **-** | - |
| DAPSA score (n = 16) | 12.0 (5.9 – 24.5) | **-** | - |
| Psoriasis Disease Duration (years) | 20 (15 – 33) | - | - |
| Total BSA Index | 4.3 (2.4 – 11.1) | **-** | - |
| **Clinical Laboratory values** |  |  |  |
| Total cholesterol (mg/dL) | 179.21 ± 27.97 | 183.85 ± 45.05 | 0.548 |
| HDL cholesterol (mg/dL) | 52 (45 – 61) | 52 (44 - 72) | 0.922 |
| LDL cholesterol (mg/dL) | 96 (83 – 118) | 104 (71 - 130) | 0.927 |
| Triglycerides (mg/dL) | 94 (77 – 136) | 93 (74 - 136) | 0.745 |
| **hs-CRP** (mg/L) | **3.5 (0.9 – 5.5)** | **1.3 (0.7 - 2.1)** | **0.003** |
| Cholesterol efflux capacity | 0.96 (0.85 – 1.09) | 1.00 (0.86 - 1.09) | 0.683 |
| **GlycA** (µmol/L) | **397 (351 – 448)** | **334 (313 - 362)** | **<0.001** |
| Hemoglobin A1C | 5.6 (5.4 – 5.8) | 5.4 (5.1 - 5.7) | 0.255 |
| **Serum cytokines (pg/mL)** |  |  |  |
| IL-1β (n = 30, 39) | 0.13 (0.04 - 0.21) | 0.06 (0.03 - 0.17) | 0.171 |
| **IL-6 (n = 31, 43)** | **1.53 (0.83 - 3.35)** | **0.83 (0.41 - 1.38)** | **<0.001** |
| IL-12/23 (n = 7, 10) | 77.88 (51.36 - 92.04) | 73.60 (38.94 - 105.00) | 0.725 |
| IL-17A (n= 31, 36) | 1.31 (0.42 - 2.39) | 0.72 (0.30 - 1.64) | 0.129 |
| TNF-α (n=31, 43) | 1.09 (0.51 - 2.19) | 1.14 (0.51 - 1.52) | 0.609 |
| **IFN-γ (n= 31, 43)** | **7.96 (5.93 - 18.89)** | **4.16 (2.72 - 7.07)** | **<0.001** |
| **CT** |  |  |  |
| Visceral Adipose Tissue (cc) | 17778 (9667 – 23002) | 12044 (7410 - 21446) | 0.196 |
| **Subcutaneous Adipose Tissue (cc)** | **19892 (14227 - 25671)** | **12376 (10004 - 18428)** | **0.023** |
| **PET-CT** |  |  |  |
| Aortic vascular (TBR) | 1.78 (1.64 - 2.00) | 1.73 (1.66 - 1.88) | 0.416 |
| **Bone Marrow (SUV_max_)** | **4.09 (3.34 – 5.38)** | **3.42 (3.10 - 4.02)** | **0.020** |
| **Liver (SUV_max_)** | **5.27 (4.29 – 6.59)** | **4.38 (3.48 - 5.03)** | **0.032** |
| Spleen (SUV_max_) | 3.81 (3.26 – 4.54) | 3.45 (3.02 - 3.71) | 0.115 |
| Subcutaneous Adipose (SUV_max_) | 0.60 (0.45 – 0.68) | 0.55 (0.46 - 0.61) | 0.462 |
| **CCTA (per artery)** | **PsA**  **(n = 117 arteries)** | **NPV**  **(n = 168 arteries)** |  |
| TB (x100), mm^2^ | 1.27 ± 0.58 | 1.14 ± 0.46 | 0.138 |
| NCB (x100), mm^2^ | 1.21 ± 0.56 | 1.10 ± 0.46 | 0.189 |

PsA, psoriatic arthritis; NPV, non-psoriatic volunteers; cDMARD, conventional disease modifying antirheumatic drugs; NSAIDs, nonsteroidal anti-inflammatory drugs; BMI, body mass index; PASI, psoriasis area severity index; BSA, body surface area; HDL, high density lipoprotein; LDL, low density lipoprotein; CRP, C-reactive protein; GlycAm glycoprotein acetylation; IL-, interleukin-; IFN, interferon; TBR, target-to-background ratio; CT, computed tomography; PET-CT, positron emission tomography; SUV_max_, maximal standardized uptake value; CCTA, coronary artery CT angiography; TB, total coronary artery burden; NCB, non-calcified coronary artery burden
