# Supplementary material for: PET/CT-Based Characterization of 18F-FDG Uptake in Various Tissues Reveals Novel Potential Contributions to Coronary Artery Disease in Psoriatic Arthritis
Source: Front Immunol. 2022 Jun 2;13:909760. doi: 10.3389/fimmu.2022.909760 (PMC9201918; doi:10.3389/fimmu.2022.909760)
Supplement: Supplementary file 2 [file Table_2.docx]

**Supplementary Table 2. Regression analysis of systemic inflammation and fat variables with NCB in biologic-naïve subjects with PsA (n = 34), adjusted for Framingham Risk Score and visceral adiposity**

| **Variable of interest** | $\boldsymbol{\beta}$ **estimate** | **Standardized** $\boldsymbol{\beta}$ **estimate** | **Partial** $\boldsymbol{R}^{\mathbf{2}}$ | **P-value** |
| --- | --- | --- | --- | --- |
| Bone marrow SUV | 0.096 | 0.304 | 7.7 | 0.098 |
| **Liver SUV** | **0.134** | **0.486** | **21.4** | **0.013** |
| **Spleen SUV** | **0.155** | **0.383** | **13.4** | **0.043** |
| **Subcutaneous Fat SUV** | **0.641** | **0.379** | **16.6** | **0.027** |
| Aortic vascular TBR | -0.168 | -0.07 | -1.8 | 0.76 |

NCB, non-calcified coronary artery burden; PsA, psoriatic arthritis; SUV, standardized uptake value; TBR, target-to-background ratio
